# Case Report: Infective endocarditis following arteriovenous graft in a peritoneal dialysis patient

**DOI:** 10.3389/fcvm.2025.1570949

**Published:** 2025-06-23

**Authors:** Fangzhong Huang, Xingzhen Zhang, Xuchun Xu, Jun Ying, Qiuping Fan, Shuangqing Li, Jian Huang

**Affiliations:** Department of Nephrology, Jinhua Municipal Central Hospital (Affiliated Jinhua Hospital, School of Medicine, Zhejiang University), Jinhua, Zhejiang, China

**Keywords:** infective endocarditis, peritoneal dialysis, arteriovenous graft, end-stage renal disease, *staphylococcus aureus*

## Abstract

Infective endocarditis (IE) is a severe and frequently fatal complication in dialysis patients, particularly those with vascular access devices such as arteriovenous grafts (AVGs) or fistulas. We describe the case of a 55-year-old male with end-stage renal disease (ESRD) on peritoneal dialysis (PD), who developed advanced IE following repeated punctures of an AVG. The patient initially presented with fever, erythema, and swelling at the graft site, which progressed to bacteremia due to *Staphylococcus aureus*. Despite completing a full course of antibiotics and undergoing both mitral and aortic valve replacement, the patient suffered recurrent episodes of acute heart failure requiring repeated hospital admissions. This rare case highlights the substantial risk of IE in dialysis patients with vascular access devices and the importance of early detection and timely intervention. The clinical difficulties and economic strain associated with IE management in this population point to the need for close surveillance, rapid diagnosis, and individualized treatment plans to improve outcomes and control healthcare expenditures.

## 1 Introduction

Infective endocarditis (IE) is a serious and often life-threatening complication in dialysis patients, particularly those using arteriovenous grafts (AVGs) or fistulas, due to the frequent need for vascular access (1–3). The incidence of IE is high in this group (3, 4), and the typical symptoms, fever, fatigue, and heart murmurs, can lead to delays in diagnosis (1). If left untreated, IE may result in severe outcomes (5), including heart failure, embolic events, and increased mortality, complicating the already difficult management of end-stage renal disease (ESRD) (6).

Diagnosing IE in dialysis patients requires a strong clinical suspicion (7). Echocardiography, particularly transesophageal echocardiography, remains the gold standard for detecting endocardial involvement, while blood cultures are essential for identifying the pathogen (8). Early diagnosis and prompt initiation of appropriate antibiotic therapy are critical, with some cases necessitating surgical intervention such as valve replacement to prevent progression (9–11). Multidisciplinary care plays a key role in guiding treatment and improving patient prognosis (6, 9).

We present a rare case involving a PD patient who developed severe IE after repeated punctures of an AVG. This complication resulted in substantial clinical deterioration and significant financial burden (2, 12, 13). The case stresses the need for early recognition and proper management of infections linked to vascular access, offering practical insights for refining diagnostic and therapeutic approaches in the dialysis population.

## 2 Case presentation

### 2.1 Medical history

A 55-year-old male was admitted on June 9, 2021, with a one-month history of intermittent fever. He had been receiving PD for 3 years. Informed consent was obtained, and the study was approved by the hospital's ethics committee. Three years prior, the patient had been hospitalized with severe lower limb edema, chest tightness, and exertional dyspnea. He was diagnosed with chronic kidney disease stage 5, renal anemia, hypertension, and hypertensive nephropathy. A PD catheter was placed on November 18, 2016, and regular PD treatment was initiated. In 2020, he experienced multiple episodes of acute heart failure due to inadequate PD. At that time, his PD regimen consisted of 2.5% glucose solution, 2,000 ml per exchange every 3–4 h, four times daily, along with an overnight dwell of 2.5% glucose solution, 2,000 ml for 8–10 h, following a continuous ambulatory PD protocol. The daily ultrafiltration volume was ∼600 ml, and he had no residual urine output. Due to poor dialysis efficiency, combined dialysis (PD + hemodialysis) was recommended. An AVG was created in his left arm on February 5, 2021, owing to poor vascular access(small vessel diameter and arterial calcification). Two months before the current admission, he developed recurrent lower limb edema and chest tightness. His condition improved with combined dialysis therapy (PD plus once-weekly hemodialysis) and treatment for heart failure. One month prior to admission, he began experiencing intermittent high-grade fever (up to 41°C), chills, swelling at the AVG site, cough with white sputum, chest tightness, nocturnal dyspnea, nausea, vomiting, fatigue, and loss of appetite. Intermittent antibiotic therapy was given, but the symptoms persisted. Two days before admission, his fever returned, reaching 40°C, again accompanied by chills. On June 7, 2021, laboratory testing revealed: white blood cell count 10.83 × 10⁹/L, neutrophils 80.3%, red blood cell count 2.88 × 10¹²/L, hemoglobin 73 g/L, and platelet count 195 × 10⁹/L. High-sensitivity C-reactive protein (hs-CRP) was significantly elevated at 151.10 mg/L. Chest computed tomography showed moderate pleural effusion in both lungs. Given the ongoing fever and unresolved symptoms, the patient was admitted to the nephrology department for further investigation and treatment.

**Figure 1:**
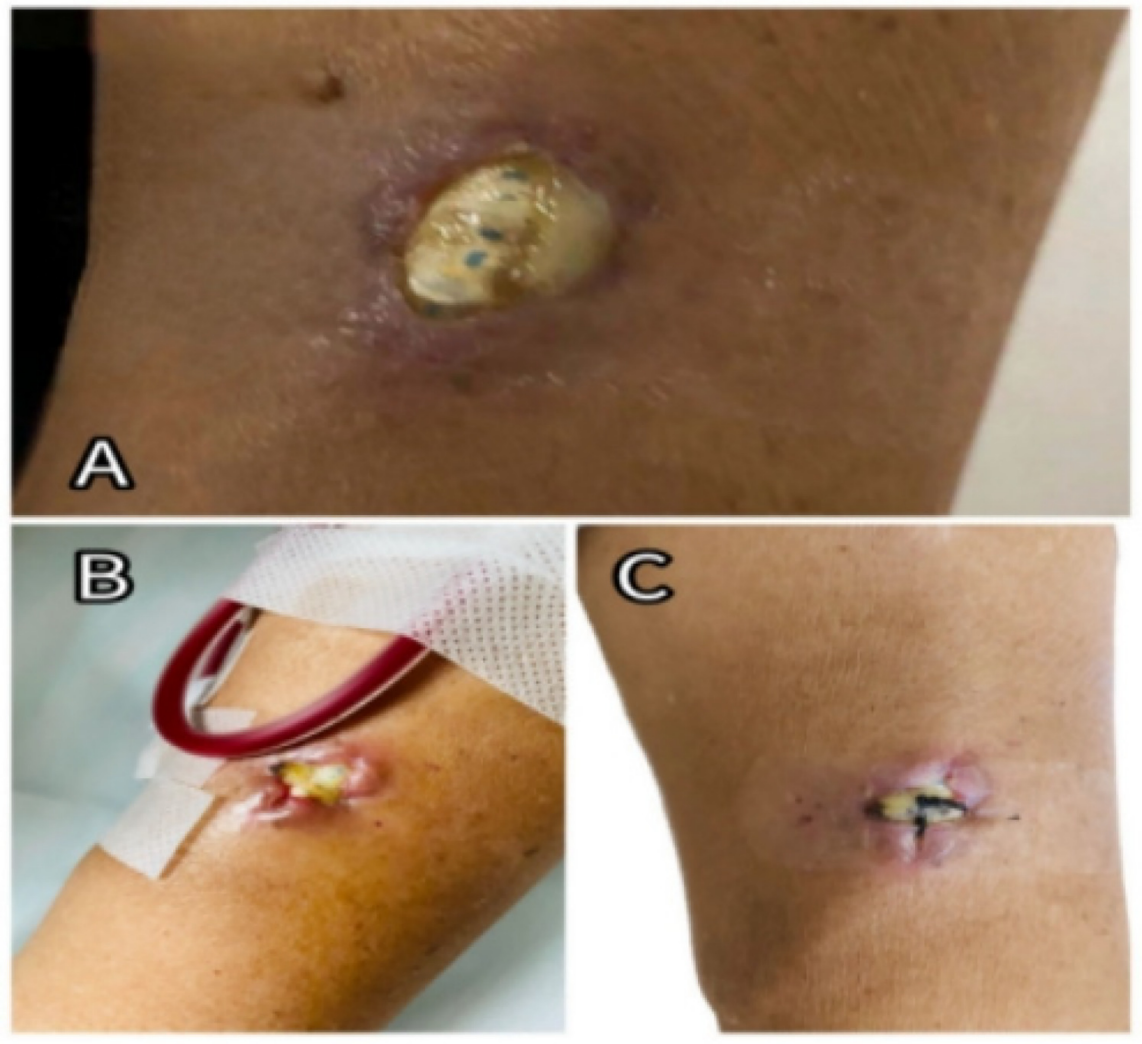
**(A)** Exposed site of the left forearm AVG at admission. **(B)** Post-suturing appearance of the ulcerated area, showing poor wound healing; sutures had been removed. **(C)** Condition of the ulcerated site following a later closure procedure.

The patient had a history of hypertension (controlled with medications), coronary artery disease, coronary stent placement, and heart failure. On physical examination, he appeared chronically ill with signs of anemia. Breath sounds were coarse, with scattered moist rales, and breath sounds were absent in the lower regions of both lungs. Cardiac auscultation revealed a holosystolic murmur at the mitral valve (Grade III–IV) and a diastolic murmur at the aortic valve (Grade III). Moderate bilateral lower limb edema was present. Examination of the left arm revealed redness, swelling, and a 2 × 1 cm ulcer, as shown in Figure 1A. The AVG in the left arm produced only a faint whooshing bruit and thrill. The PD catheter was in place and secure. Admission diagnoses included: infectious fever, acute heart failure (NYHA class III), chronic kidney disease stage 5, current hemodialysis and PD treatment, renal anemia, coronary artery disease, post-coronary stent placement, hypertensive nephropathy, and secondary hyperparathyroidism.

### 2.2 Diagnosis and treatment course

**Figure 2:**
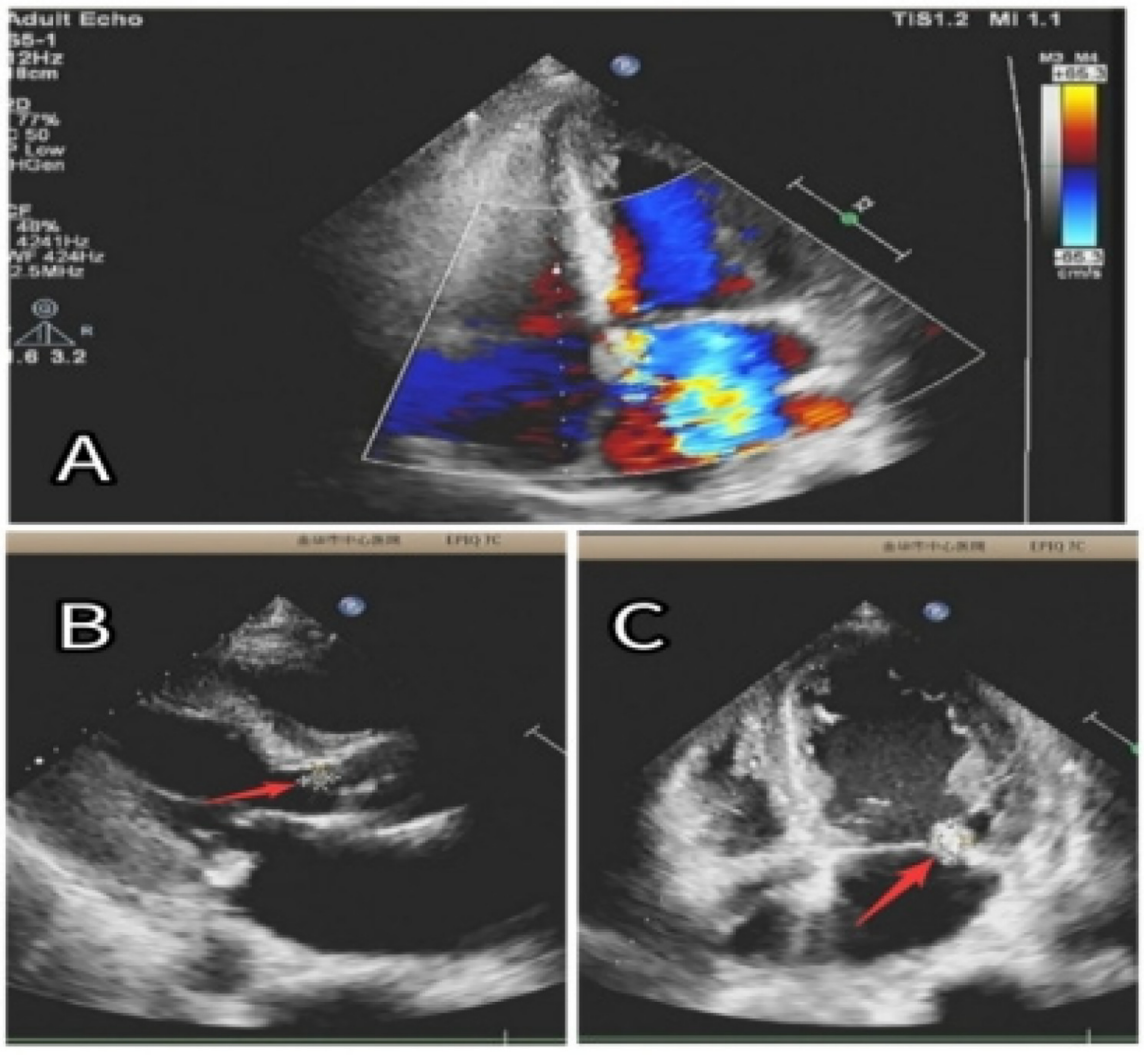
**(A)** Significant regurgitation in both the mitral and aortic valves. **(B)** An 8 × 6 mm vegetation (red arrow) was seen on the aortic valve. **(C)** A 15 × 10 mm vegetation (red arrow) was identified on the posterior leaflet of the mitral valve.

Given the suspicion of an AVG infection and respiratory symptoms (coughing with a small amount of yellow purulent sputum) upon admission, empirical anti-infective therapy was initiated with vancomycin (0.5 g, intravenous infusion, once every 3 days) and cefoperazone-sulbactam (1.5 g, intravenous infusion, every 12 h), covering both Gram-positive and Gram-negative bacteria. The patient continued regular PD on the remaining 6 days of the week, with once-weekly hemodialysis on Tuesdays. He was also prescribed clopidogrel hydrogensulfate (75 mg once daily) for antiplatelet therapy and atorvastatin calcium (20 mg once daily) for vascular plaque stabilization. On June 11, the infected AVG was surgically removed under local anesthesia, and cultures were obtained. *Staphylococcus aureus* was identified from both the AVG tissue and blood cultures. On June 12, echocardiography showed new vegetations on the mitral and aortic valves, leading to moderate-to-severe regurgitation (as shown in Figure 2). Based on clinical signs, blood culture results, and echocardiographic findings, a diagnosis of IE secondary to AVG infection was made. Following consultation with specialists in cardiology, cardiothoracic surgery, and infectious diseases, a multidisciplinary team recommended continuing intravenous antibiotics for 4–6 weeks prior to considering valve replacement surgery (5, 10).

Later that day, the patient was transferred to the intensive care unit due to septic shock and acute heart failure. He underwent continuous renal replacement therapy via a right femoral venous catheter, as well as bilateral thoracentesis with drainage. Anti-infective therapy continued throughout. After clinical stabilization, he was transferred back to the general ward on June 17. The femoral vein catheter was removed, and regular PD was resumed. Antibiotics were changed to piperacillin-tazobactam (4.5 g, intravenous infusion, every 12 h) and linezolid (600 mg orally every 12 h).

By June 25, the patient's condition had improved significantly. Vital signs were stable, and inflammatory markers had decreased (as shown in Table 1). He was discharged with the following treatment plan: linezolid (600 mg orally every 12 h), polysaccharide iron (300 mg daily), metoprolol succinate (47.5 mg daily), clopidogrel hydrogensulfate (75 mg daily), atorvastatin (20 mg once daily), esomeprazole (20 mg daily before meals), and PD using 2.5% glucose solution (2,000 ml every 4 h, no overnight dwell).

**Table 1:** Summary of key clinical indicators during hospitalization.

Indicator	6–9	6–11	6–12	6–13	6–14	6–16	6–19	6–25
Temp (°C)	39	38	37	37.1	37.3	37.2	37.1	37
BP (mmHg)	108/57	104/50	108/51	112/63	116/67	125/54	135/50	148/67
Alb (g/L)	32	30	33	32	27	28	31	33
Hb (g/L)	72	61	66	84	65	74	79	91
hs-CRP (mg/L)	198	164	141	129	118	59	31	28
PCT (ng/ml)	12.55	8.57	7.97	4.82	2.97	1.73	1.15	0.78
TnI (μg/L)	0.26	0.96	0.99	1.74	2.14	1.99	1.48	0.43
NT-pro BNP (g/ml)	19,600	34,000	51,300	58,000	35,900	27,000	34,300	35,000

6–9: on June 9, 2021, with subsequent dates following the same pattern. Temp, temperature; BP, blood pressure; Alb, albumin; Hb, hemoglobin; hs-CRP, high-sensitivity C-reactive protein; PCT, procalcitonin; TnI, troponin I; NT-pro BNP, N-terminal pro B-type natriuretic peptide.

### 2.3 Follow-up summary

**Figure 3:**
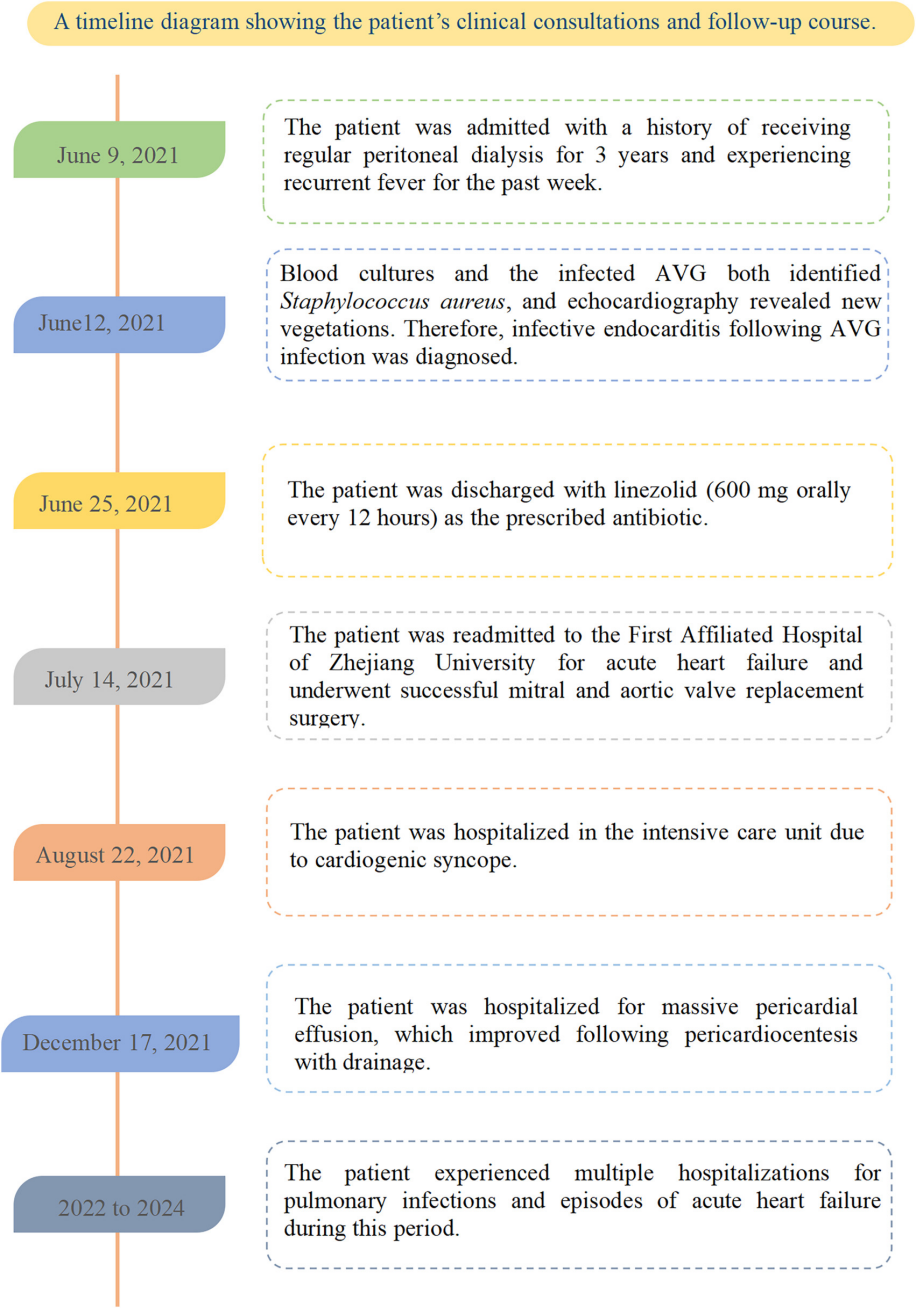
A timeline diagram showing the patient's clinical consultations and follow-up course.

The patient was readmitted on July 14, 2021, due to acute heart failure and was discharged on July 20 to undergo mitral and aortic valve replacement surgery at the First Affiliated Hospital of Zhejiang University. Postoperatively, he was started on warfarin for anticoagulation. On August 22, he was hospitalized in the intensive care unit for cardiogenic syncope and discharged after stabilization. On December 27, he required treatment for a large pericardial effusion, which resolved following pericardiocentesis with drainage. Between 2022 and 2024, the patient was hospitalized multiple times for pulmonary infections and episodes of acute heart failure. He was admitted to our department on the following dates: May 22, 2022; July 28, 2022; November 28, 2022; February 17, 2023; April 9, 2023; August 11, 2023; January 24, 2024; July 7, 2024; and December 8, 2024 (as shown in Figure 3). In June 2024, a new AVG was placed in the right forearm, followed by two balloon angioplasties to treat AVG thrombosis.

At present, the patient continues PD using a nighttime dry abdomen protocol, with adjustments based on his clinical status. The current regimen includes low-calcium PD solution (2,000 ml every 3–4 h, 4–5 times daily, yielding ∼700 ml ultrafiltration, with no urine output). Medications include atorvastatin (20 mg daily), warfarin (0.625 mg daily), felodipine (5 mg every morning), and sacubitril-valsartan (100 mg twice daily).

## 3 Discussion

IE is a serious complication in dialysis patients, driven by a combination of interconnected risk factors (4). A major contributor is the frequent use of central venous catheters and AVGs for hemodialysis access, which serve as direct pathways for bacterial entry into the bloodstream and substantially increase the risk of bacteremia (3). Mechanical stress and repeated handling of these devices can cause localized infections, which may progress to IE. The dialysis process itself plays a role in infection susceptibility. Hemodialysis can cause microvascular injury and disrupt endothelial integrity, making it easier for bacteria to adhere and colonize. Although PD is generally less invasive, it still carries a risk of infection due to potential microbial contamination during exchanges. Chronic exposure to dialysis over time impairs both local and systemic immune responses, reducing the body's ability to fight infections effectively (14). In addition, comorbidities common among dialysis patients, such as diabetes mellitus, hypertension, and cardiac disease, further weaken immune defenses. The use of immunosuppressive medications to manage autoimmune or inflammatory conditions can also impair host resistance to infection. Other patient-specific factors, including advanced age, prolonged dialysis duration, and poor nutritional status, increase vulnerability. Chronic dialysis combined with impaired wound healing and fragile tissue integrity raises the likelihood of infections reaching the cardiac valves. These overlapping elements reflect the complex etiology of IE in dialysis patients and emphasize the need for close monitoring, timely diagnosis, and targeted antimicrobial therapy to reduce the risk of this life-threatening condition (15).

In this case, multiple factors contributed to the development of infective endocarditis. The AVG was likely implanted too superficially, and repeated punctures during hemodialysis caused localized ulceration and infection (as shown in Figure 1), which became the primary source of bacteremia. The patient had also been in a compromised state for the preceding month, with persistent fever, malnutrition, anemia, hypotension, and a calcium-phosphorus imbalance (as shown in Table 1). Additionally, long-term peritoneal dialysis and a history of coronary artery disease with prior stent placement led to immune suppression and increased susceptibility of the heart valves to infection.

This case illustrates the complex challenges of managing a PD patient who developed severe complications following an AVG infection that progressed to IE. Several important clinical lessons can be drawn. First, the decision to place an AVG in February 2021 may have been premature. Since PD was still providing acceptable renal replacement with adjustments to the regimen throughout the treatment course, the necessity of initiating AVG access was questionable. In patients where PD remains effective, especially those with comorbidities such as malnutrition, anemia, and calcium-phosphate imbalance, the timing of AVG creation should be weighed carefully, as these factors increase infection risk. Second, the case highlights the importance of individualized dialysis protocols. Although the patient was initially on a standard continuous ambulatory PD regimen, dialysis adequacy was not optimal. Adjusting the dialysate concentration and dwell time in response to the patient's condition, including ongoing infection, cardiac status, and metabolic abnormalities, is essential to improving dialysis performance and reducing complications. Moreover, delayed recognition of infection at the AVG site contributed to disease progression. Intermittent fever and localized signs such as redness should have prompted earlier investigation. Timely identification of infection and prompt initiation of antibiotics might have prevented the escalation to valve involvement and subsequent heart failure. This case also reflects the poor long-term prognosis and high treatment burden associated with IE in ESRD patients. Despite undergoing valve replacement surgery and receiving prolonged antibiotic therapy, the patient continued to face recurrent admissions for heart failure and pulmonary infections. These outcomes point to the need for preventive measures, including early detection of access-related infections, better dialysis management, and active control of comorbidities.

In summary, this case reinforces the importance of personalized care in ESRD patients. Timely responses, skilled AVG handling, early treatment of vascular access infections, tailored PD strategies, and integrated management of comorbid conditions are all essential for reducing complications and improving outcomes in this high-risk group.

## 4 Conclusion

This PD patient developed skin ulceration at the AVG site, which led to a *Staphylococcus aureus* bloodstream infection and severe infective endocarditis. Although he completed antibiotic treatment and underwent mitral and aortic valve replacement, he continued to experience repeated hospitalizations due to acute heart failure. This case highlights the importance of individualized care in ESRD patients. Early detection and prompt management of vascular access site infections, careful adjustment of dialysis regimens, and thorough management of comorbid conditions are critical to improving outcomes and reducing complications in this high-risk group.

## Data Availability

The original contributions presented in the study are included in the article/Supplementary Material, further inquiries can be directed to the corresponding author.
